# Protein Adsorption on Various Plasma-Treated Polyethylene Terephthalate Substrates

**DOI:** 10.3390/molecules181012441

**Published:** 2013-10-10

**Authors:** Nina Recek, Morana Jaganjac, Metod Kolar, Lidija Milkovic, Miran Mozetič, Karin Stana-Kleinschek, Alenka Vesel

**Affiliations:** 1 Jozef Stefan International Postgraduate School, Jamova 39, Ljubljana 1000, Slovenia; E-Mails: nina.recek@ijs.si (N.R.); metod.kolar@ijs.si (M.K.); 2 Anti Doping Laboratory Qatar, Doha, 27775, State of Qatar; E-Mails: mjaganjac@adlqatar.com or morana.jaganjac@irb.hr; 3 Rudjer Boskovic Institute, Bijenicka 54, Zagreb 10000, Croatia; E-Mails: lidija.mrakovcic@irb.hr; 4 Plasma Laboratory, Institute Jozef Stefan, Jamova 39, Ljubljana 1000, Slovenia; E-Mail: miran.mozetic@guest.arnes.si; 5 Laboratory for Characterization and Processing of Polymers, Faculty of Mechanical Engineering, University of Maribor, Smetanova 17, Maribor 2000, Slovenia; E-Mail: karin.stana@uni-mb.si

**Keywords:** oxygen and fluorine plasma treatment, polymer surface modification, protein adsorption, cell adhesion, quartz crystal microbalance (QCM)

## Abstract

Protein adhesion and cell response to plasma-treated polymer surfaces were studied. The polymer polyethylene terephthalate (PET) was treated in either an oxygen plasma to make the surface hydrophilic, or a tetrafluoromethane CF_4_ plasma to make the surface hydrophobic. The plasma source was radiofrequency (RF) discharge. The adsorption of albumin and other proteins from a cell-culture medium onto these surfaces was studied using a quartz crystal microbalance (QCM), X-ray photoelectron spectroscopy (XPS) and atomic force microscopy (AFM). The cellular response to plasma-treated surfaces was studied as well using an MTT assay and scanning electron microscopy (SEM). The fastest adsorption rate was found on the hydrophilic oxygen plasma-treated sample, and the lowest was found on the pristine untreated sample. Additionally, the amount of adsorbed proteins was higher for the oxygen-plasma-treated surface, and the adsorbed layer was more viscoelastic. In addition, cell adhesion studies support this finding because the best cell adhesion was observed on oxygen-plasma-treated substrates.

## 1. Introduction

Polymers are often used in various biomedical applications for medical implants, for tissue engineering and for therapeutic purposes, e.g., for studying cancer metastasis [1,2,3]. In these cases, the polymers interact with blood proteins or cells. The first interaction involves water molecules, followed by proteins [1]. If there are many proteins, competitive adsorption occurs. The adsorbed protein film can show time-dependent conformational changes, which may cause desorption or even protein exchange [2]. Protein adsorption is very complex because it is driven by various forces, including Van der Waals, hydrophobic and electrostatic forces. The adsorbed protein layer has an important influence on cell adhesion, which is the last step in the interaction between the polymer and the host environment. Therefore, knowledge about protein adsorption and conformation is important for explaining cell-surface interactions. 

A gaseous plasma is often used for surface modifications of polymers to improve their biocompatibility [4,5,6,7,8,9,10] or to improve resistance to bacterial infections [11,12,13,14]. Various plasmas can be used to achieve the desired effect. Oxygen plasma can be used to introduce polar functional groups to the surface and to make the surface hydrophilic, while tetrafluoromethane plasma can be used to introduce non-polar functional groups and to make the surface hydrophobic [15,16]. In addition, the surface charge can be changed after plasma treatment. Plasma treatment can enhance cell adhesion, but less is known about protein adhesion on plasma-treated surfaces [17,18].

Proteins contain various regions, which can be hydrophilic/hydrophobic, charged/uncharged and polar/non-polar. Thus, at hydrophilic interfaces, proteins predominantly expose hydrophilic residue-containing patches toward the surface, and on hydrophobic surfaces, proteins direct their hydrophobic patches to the surface [19]. Analogously, proteins adsorbing at positively or negatively charged interfaces tend to expose the oppositely charged regions to the surface [19]. Proteins tend to adhere more strongly to the following: non-polar surfaces (relative to polar surfaces), areas of high surface tension (relative to areas of low surface tension) and charged substrates (relative to uncharged substrates) [19]. Non-polar surfaces can destabilise proteins and thereby facilitate conformational reorientations that lead to strong inter-protein and protein–surface interactions [20]. Thus, many researchers report that proteins have a higher affinity to hydrophobic surfaces and a lower affinity to hydrophilic surfaces [21]. An example of the possibly misleading nature of this general rule is the adsorption of glycoproteins, which adsorb extensively on hydrophilic surfaces and sparsely on hydrophobic surfaces [19].

Protein adsorption can be studied using a quartz crystal microbalance with a dissipation unit (QCM-D) [22,23,24,25,26]. This is one of few techniques that can be used for a direct observation of the adsorption process because it enables real-time measurements. Furthermore, QCM is a high-resolution mass-sensing technique with a sensitivity in the range of nanograms per cm^2^ [23]. QCM measures the mass per unit area by measuring the change in frequency of a quartz crystal. Any adsorption/desorption of mass from the quartz sensor causes a change in the resonant frequency. A QCM that is equipped with a dissipation unit can also provide important information about the structure (rigidity) of the adsorbed layer. In the past decade, interest in using this technique for studying interactions between biological material and substrates and for monitoring cell adhesion has increased.

The aim of this study was to determine how different surface modifications of a PET polymer using low-pressure plasma treatments influence the adsorption of albumin and other proteins from a cell culture medium. The adsorption was studied using the QCM-D technique, XPS and AFM. The results of the protein adhesion were further supported by cell adhesion studies using MTT assay [27]. The results are organised as follows. The first section describes the surface characteristics of plasma-treated samples. In the second section, we examine the protein adhesion using the XPS and AFM methods. In the third section, we describe the detailed adsorption kinetics of proteins measured with QCM, and in the last section, we introduce the cell adhesion results.

## 2. Results and Discussion

### 2.1. Surface Characterisation of Plasma-Treated Samples

**Figure 1:**
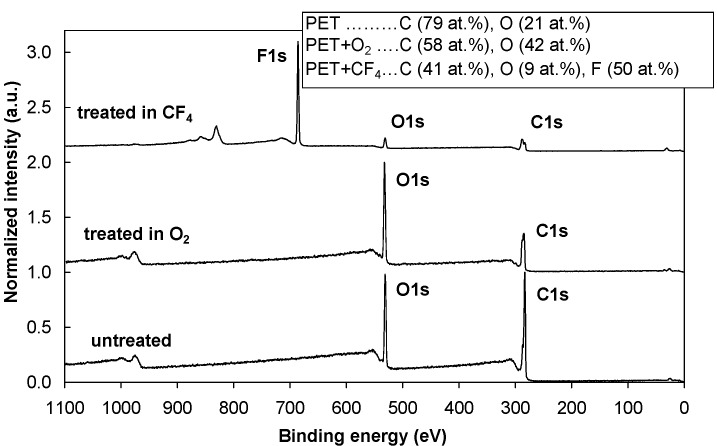
XPS survey spectra for the control (untreated) PET sample and for PET samples treated in O_2_ and CF_4_ plasma.

**Figure 2:**
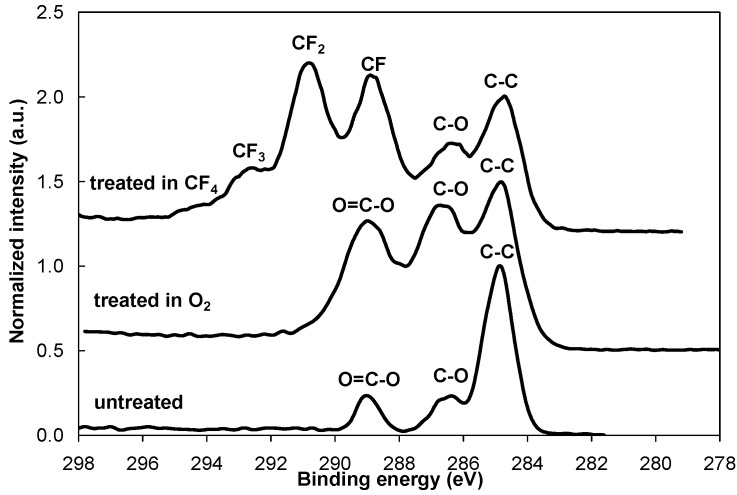
XPS high-resolution spectra of carbon C1s for the control PET sample and for the PET samples treated in O_2_ and CF_4_ plasma.

Figure 1 shows an XPS survey spectrum for the control (untreated) PET sample and the PET samples treated in oxygen (O_2_) and tetrafluoromethane (CF_4_) plasmas. After the oxygen plasma treatment, the oxygen concentration on the surface doubled. Treating the polymer in CF_4_ plasma significantly reduced the oxygen concentration, and approximately 50 atomic % of fluorine is found. The incorporation of oxygen and fluorine species into the polymer surface during the plasma treatment resulted in the formation of different functional groups. As shown in Figure 2, the intensity of the peaks from C–O and O=C–O groups increased after the oxygen plasma treatment. Different fluorine groups are observed for the CF_4_ plasma treatment. The majority are CF_2_ groups, followed by CF groups and CF_3_ in smaller concentrations. Our results for both plasma treatments are consistent with previously published results [15,16,28,29,30,31]. The functional groups formed after treatments also alter the surface wettability. Therefore, O_2_ plasma treatment resulted in a very hydrophilic surface with a contact angle of only a few degrees, while CF_4_ plasma treatment resulted in the formation of a hydrophobic surface with a contact angle of 110°. 

### 2.2. Surface Characterisation of the Adsorbed Protein Layer

**Figure 3:**
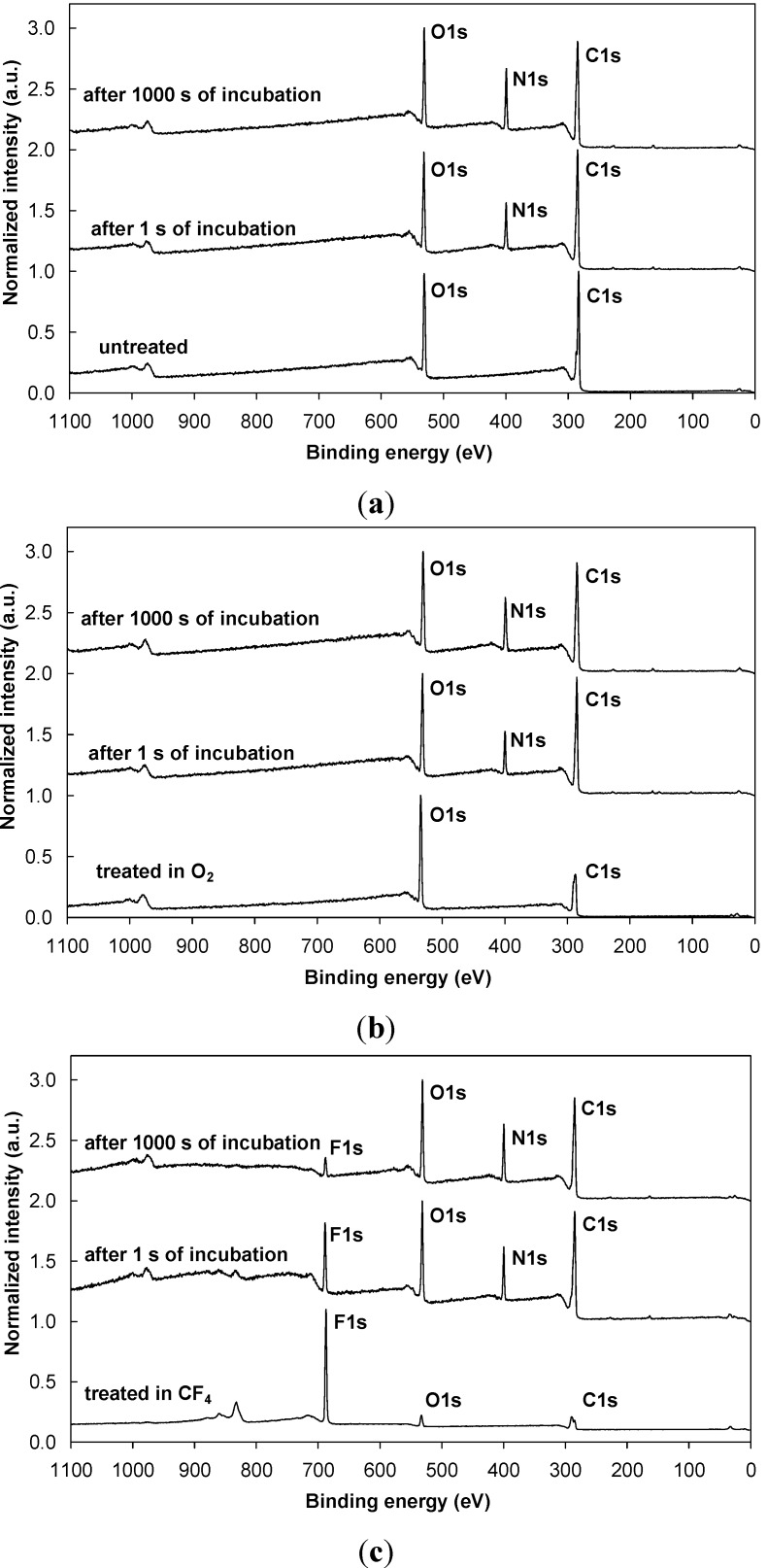
XPS survey spectrum for: (**a**) the control (untreated) PET sample before and after incubation for 1 s and 1,000 s; (**b**) the PET sample treated in O_2_ plasma before and after incubation for 1 s and 1,000 s and (**c**) the PET sample treated in CF_4_ plasma before and after incubation for 1 s and 1,000 s in albumin solution.

**Figure 4:**
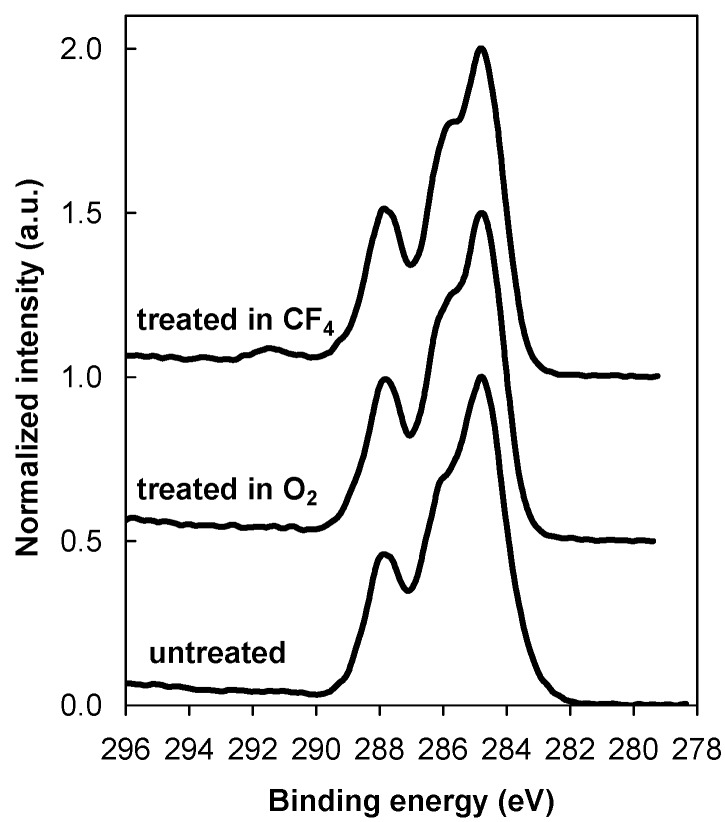
XPS high-resolution spectra of carbon C1s for the untreated sample and both plasma-treated samples after incubation in an albumin solution for 1,000 s.

Plasma-treated samples were incubated in various protein solutions, *i.e*., a pure albumin (ALB) solution and a protein mixture used as a cell culture medium (FCS/DMEM), for different periods: 1, 10, 100 and 1,000 s. Figure 3 shows typical XPS survey spectra for albumin adsorption for the shortest (1 s) and the longest (1,000 s) incubation times. Similar spectra were also observed for the FCS/DMEM solution. Figure 3a shows typical spectra for albumin adsorption on the untreated sample (control sample), while Figure 3b,c show the spectra for the samples treated in O_2_ and CF_4_ plasma, respectively. Figure 3a clearly shows the formation of a new peak after the incubation of the untreated sample in a protein solution; this peak is caused by the nitrogen that originates from the adsorbed albumin layer, regardless of whether the sample was exposed to the protein solution for 1 s or 1,000 s. Results similar to those obtained for the control samples were also observed for the plasma-treated samples—*i.e*., a nitrogen peak appeared as a result of the adsorbed protein. Supplementing the information in Figure 3, Figure 4 shows the high-resolution XPS spectra of the carbon peak recorded from the untreated sample and both plasma-treated samples after 1,000 s of incubation. Figure 4 shows that there is no pre-treatment difference between the various samples. In all cases, the shape of the spectrum is similar and typical for proteins and shows the presence of peptide and amine groups. Comparing Figure 4 with Figure 2 indicates that after 1,000 s of incubation, a layer of protein almost completely masks the plasma-treated substrate, because functional groups from the plasma-treated substrate are no longer visible. 

**Figure 5:**
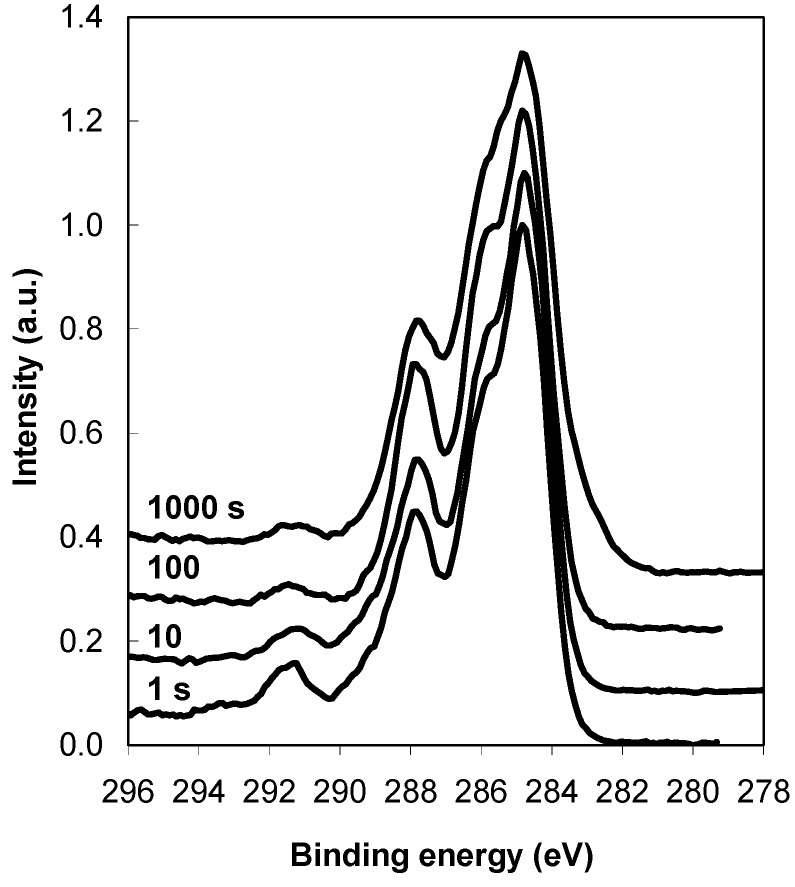
XPS high-resolution spectra of carbon C1s for the CF_4_ plasma-treated PET sample after incubation in an albumin solution for various periods.

Figure 3 shows another interesting phenomenon. The CF_4_ plasma-treated sample still contains a fluorine peak that originates from the plasma-treated layer (Figure 3c). This finding indicates that the adsorbed protein layer is thinner than the detection depth of XPS. Supplementing the information in Figure 3c and Figure 5a compares the high–resolution carbon peaks for various incubation times for the CF_4_ plasma-treated PET sample. In addition to the typical protein peaks, another small peak is positioned near the 292 eV binding energy. This peak is due to the CF_2_ groups from the interface between the protein coating and the plasma-treated substrate (this peak was the most significant on the plasma-treated sample shown in Figure 2). Thus, in agreement with the XPS survey spectra (Figure 3c), we can therefore also observe traces of fluorine peaks in the high-resolution spectra shown in Figure 5.

From the intensity of the fluorine signal (Figure 3c), we can roughly estimate the protein layer thickness for the CF_4_ plasma-treated sample. The fluorine signal (*I*_*F*_0__) from the interface between the polymer substrate and the protein layer is attenuated by passing through a protein layer with a thickness *d*, as described in the following relation [21]:

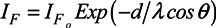
(1)
where *I_F_* is the measured fluorine signal emerging through the protein layer, *I*_*F*_0__ is the measured fluorine signal of plasma-treated sample without the protein layer, *λ* is the inelastic mean free path for F1s electrons passing through the protein layer (~3 nm), and *θ* is the electron take-off angle (45°). After rearranging Equation (1), the protein thickness can be calculated from the following:

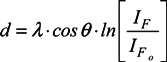
(2)


The protein thickness was calculated for all incubation times and is shown in Table 1, along with the fluorine concentration and F/C ratio. A lower fluorine signal indicates a thicker protein layer (because some of the signal was lost when passing through the protein layer). Table 1 shows that the protein thickness first increases with incubation time and later becomes constant. These results refer only to the case of the fluorine plasma-treated sample. For the oxygen plasma-treated sample, it is not possible to calculate the layer thickness because the oxygen originates not only from the polymer but also from the overlying protein. Therefore, additional information about the quantity of adsorbed proteins can be obtained only by QCM.

**Table 1:** Fluorine concentration and calculated protein film thickness *versus* incubation time in albumin solution for the CF_4_ plasma-treated PET sample.

*t* (s)	F (at.%)	F/C	*d* (nm)
1	8.4	0.14	1.9
10	4.4	0.07	4.2
100	2.8	0.04	5.3
1000	2.7	0.04	5.3

**Figure 6:**
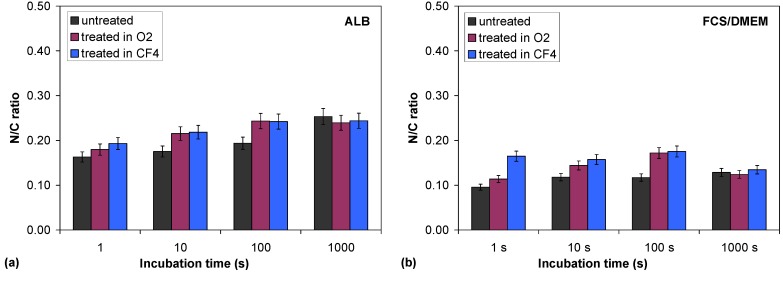
Comparison of the N/C ratio after incubation of untreated PET samples and O_2_ and CF_4_ plasma-treated samples in albumin solution (**a**) and FCS/DMEM solution (**b**).

Furthermore, to find additional information about albumin adhesion, the intensity of the nitrogen N1s peak was measured because the nitrogen peak originates only from the adsorbed layer of the protein albumin and not from the polymer substrate. The N/C ratio was calculated for the various samples, and the results are shown in Table 2 and Figure 6a. The adsorption of albumin to the polymer surfaces starts very quickly because after just 1 second there is already a substantial amount of nitrogen on the surface. With increasing incubation time, the nitrogen concentration is slightly increased.

**Table 2:** Surface composition of the samples after incubation in albumin solution (in atomic %).

PET ctrl	C	H	O	S	F	N/C
1 s	67.2	11.0	21.3	0.6	/	0.16
10 s	69.8	12.2	17.5	0.5	/	0.18
100 s	66.6	12.9	20.1	0.5	/	0.19
1000 s	63.8	16.2	19.3	0.8	/	0.25
**PET + O_2_**	**C**	**N**	**O**	**S**	**F**	**N/C**
1 s	66.1	12.0	21.3	0.6	/	0.18
10 s	64.3	13.8	21.3	0.6	/	0.22
100 s	63.6	15.5	20.2	0.7	/	0.24
1000 s	63.6	15.2	20.5	0.7	/	0.24
**PET + CF_4_**	**C**	**N**	**O**	**S**	**F**	**N/C**
1 s	61.4	11.9	17.7	0.7	8.4	0.19
10 s	61.9	13.5	17.7	0.6	4.4	0.22
100 s	61.8	15.0	19.8	0.7	2.8	0.24
1000 s	61.9	15.1	19.7	0.7	2.7	0.24

Careful examination of Figure 6a shows differences between the untreated and the plasma-treated samples, e.g., the nitrogen content is lower in the untreated sample in the first 100 s of relative to that in both plasma-treated samples (hydrophilic and hydrophobic ones). This difference in nitrogen content disappeared after 1,000 s of incubation—the nitrogen content on the polymer surface was then practically the same for all samples regardless of whether they were treated in the plasma. The nitrogen content differs only in the first few minutes of incubation; therefore, the protein adsorption is slightly faster on the two plasma-treated samples than on the untreated sample, but this difference vanished later. The protein adhesion trend for the FCS/DMEM protein mixture (Figure 6b) was similar to that of albumin. In addition, we can find more nitrogen on the surface of the plasma-treated samples in the first 100 s of incubation, while this difference later diminishes.

These results are also supported by QCM measurements, as shown later in the text. The results clearly show that proteins very quickly reach and adsorb on the polymer surface. This adsorbed layer of proteins then governs the further adhesion of cells that appear much later.

**Figure 7:**
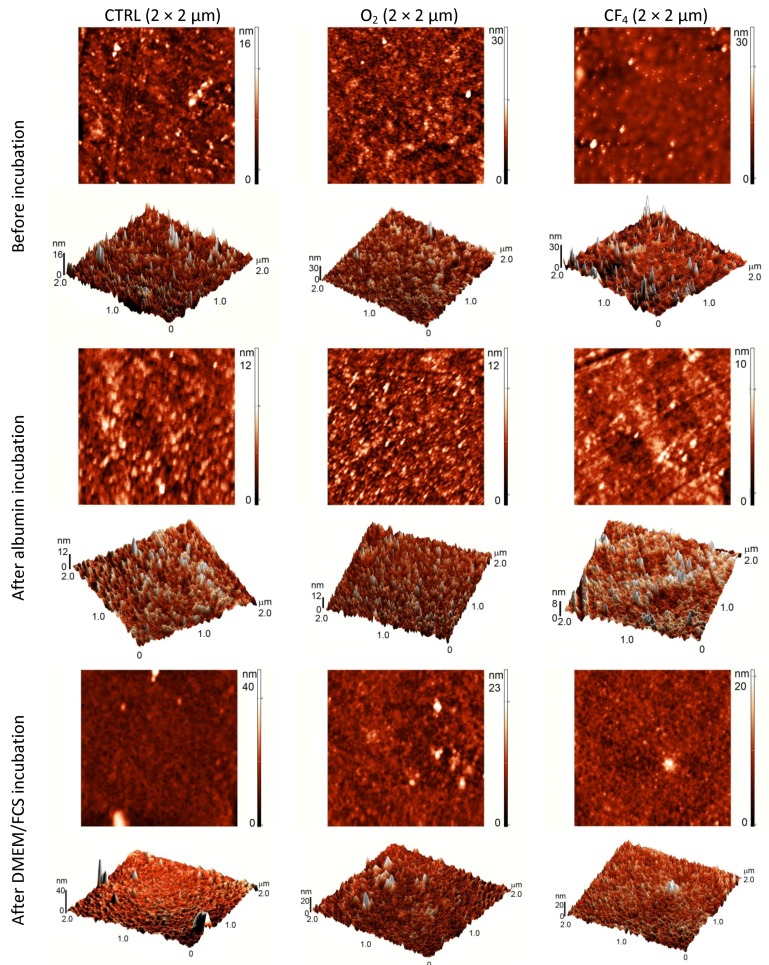
AFM images (2 × 2 µm) of different plasma-treated surfaces before and after incubation in a protein solution.

To obtain additional information regarding the surface characteristics of the plasma-treated samples, AFM analyses were performed to determine the surface topography, which can also influence protein and cell adhesion. The AFM images are shown in Figure 7. 

After oxygen plasma treatment, the surface has a highly oriented structure and is rougher compared with the control sample. The fluorine plasma-treated surface does not show any oriented structure, but several peaks are observed. After incubation in albumin, the samples do not show any significant difference in surface morphology. By contrast, after incubation in FCS/DMEM solution, the surface becomes smoother except from unevenly distributed features. These isolated peaks formed upon incubation might be protein agglomerates.

### 2.3. Adsorption Kinetics of Proteins Studied by QCM

**Figure 8:**
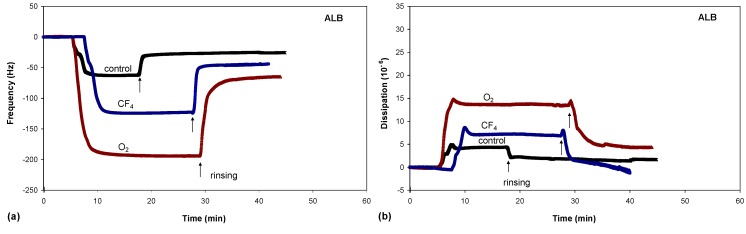
Frequency changes (**a**) and dissipation changes (**b**) for albumin adsorption on untreated and plasma-treated PET substrates.

**Figure 9:**
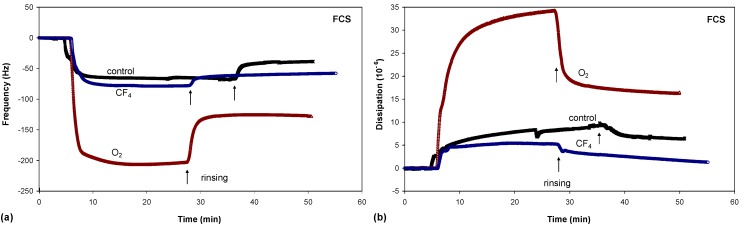
Frequency changes (**a**) and dissipation changes (**b**) for FCS adsorption on untreated and plasma-treated PET substrates.

**Figure 10:**
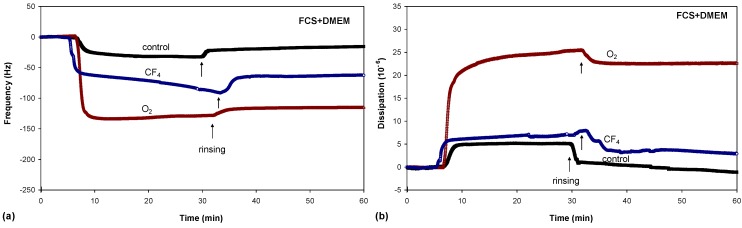
Frequency changes (**a**) and dissipation changes (**b**) for FCS/DMEM adsorption on untreated and plasma-treated PET substrates.

One of the best methods for studying the adsorption kinetics of proteins is QCM, which measures the mass per unit area by measuring the change in frequency of a quartz crystal resonator. Typical adsorption curves, *i.e*., change in frequency as a function of time, for albumin are shown in Figure 8a, while the adsorption curves for FCS and FCS/DMEM are shown in Figure 9a and Figure 10a, respectively. 

There were significant differences in protein adsorption on the plasma-treated PET surfaces. The results presented in Figure 8, Figure 9 and Figure 10 show that the protein adsorption is very fast at the beginning and later slows down, slowly reaching equilibrium when the surface becomes saturated. The adsorption is fastest for the oxygen plasma-treated PET sample; by contrast, the control sample and the fluorine plasma-treated sample show no important differences. This finding is true for all three protein solutions used. 

Another conclusion can be drawn from Figure 8, Figure 9 and Figure 10. In the case of albumin, the adsorption equilibrium was reached at Δ*f* = −63 Hz for the control sample, Δ*f* = −124 Hz for the fluorine plasma-treated sample and Δ*f* = −194 Hz for the oxygen plasma-treated sample. This frequency difference between the samples indicates that the mass of adsorbed proteins was lowest on the control sample and highest on the oxygen plasma-treated sample. By contrast, for the fluorine plasma-treated sample, the adsorbed mass was between these two values. The same results were also observed for FCS and for FCS with DMEM–in all cases, more proteins adsorbed on the O_2_ plasma-treated surface. 

After the samples were rinsed with PBS solution (the starting point of rinsing is marked with arrows in Figure 8, Figure 9 and Figure 10), the resonant frequency changes, indicating the desorption of loosely bound proteins. The desorption is the most pronounced for the control sample, for which the majority of the proteins was desorbed. The plasma-treated samples also showed high desorption, but because of the higher mass of pre-adsorbed proteins, many proteins remain on the surface in comparison to the control sample. In particular, the oxygen plasma showed the most favourable conditions; *i.e*., the majority of the proteins remained on the surface, and we probably have several monolayers of protein, which was further proved with dissipation measurements. These results therefore show that more proteins were adsorbed on the hydrophilic (polar) surface than on the hydrophobic (non-polar) one. 

**Figure 11:**
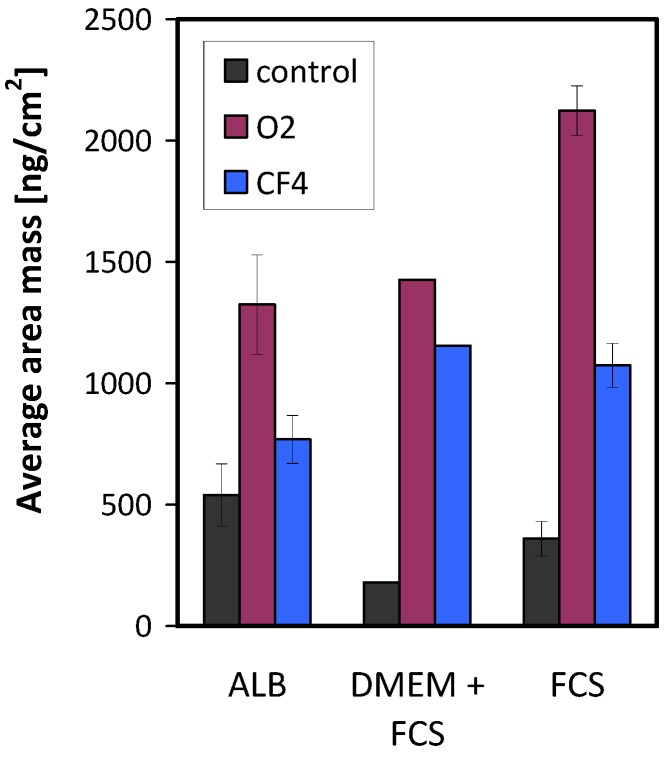
Mass of adsorbed proteins on untreated and plasma-treated samples.

The mass of the adsorbed proteins on the various plasma-treated samples is shown in Figure 11. Again, all protein solutions show a similar result: the largest amount of adsorbed proteins was on the oxygen plasma-treated surface, and the lowest amount was found on the surface of the control (untreated) sample. 

**Figure 12:**
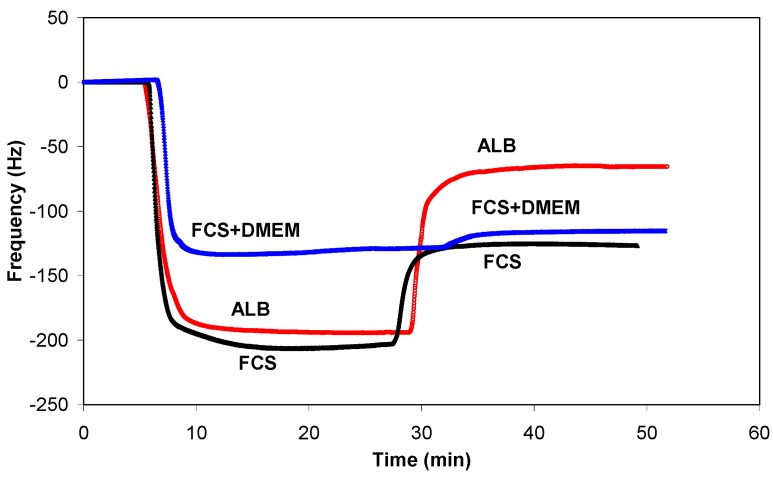
Comparison of the adsorption process of different protein solutions to the oxygen plasma-treated surface.

Another finding is the huge change in the frequency following rinsing in the case of albumin and FCS; by contrast, in the case of FCS with the addition of DMEM, the difference is very small (desorption following rinsing is negligible). Figure 12 presents this finding more clearly and shows only the adsorption curves for all three protein solutions on the surface of the oxygen plasma-treated samples. The situation for the control sample or the sample treated in CF_4_ plasma is similar to that for oxygen. The presence of DMEM appears to affect protein adsorption kinetics. One important component of DMEM is salt; the type of salt can vary, and the salt is a source of various ions. Furthermore, as reported in the literature, dissolved ions (*i.e*., the ionic strength) can change the protein adsorption process [19]. This is probably the reason for more stable adsorption of proteins on the polymer surface with FCS compared with the surface with DMEM.

In conjunction with the measured QCM frequency, dissipation measurements can provide further information regarding protein adsorption (Figure 8b, Figure 9b and Figure 10b). The change in dissipation was smaller for the CF_4_ plasma-treated sample than for the O_2_ plasma-treated sample. The lowest dissipation was found for the control sample, except for FCS adsorption (Figure 9b). The dissipation was the largest for the oxygen plasma-treated sample; therefore, the adsorbed protein layer was thicker and less compact than that on the CF_4_ plasma-treated sample or the control sample. This difference can also be caused by the different conformation of proteins on the surface or the binding/trapping of more water molecules in the adsorbed protein film [26].

**Figure 13:**
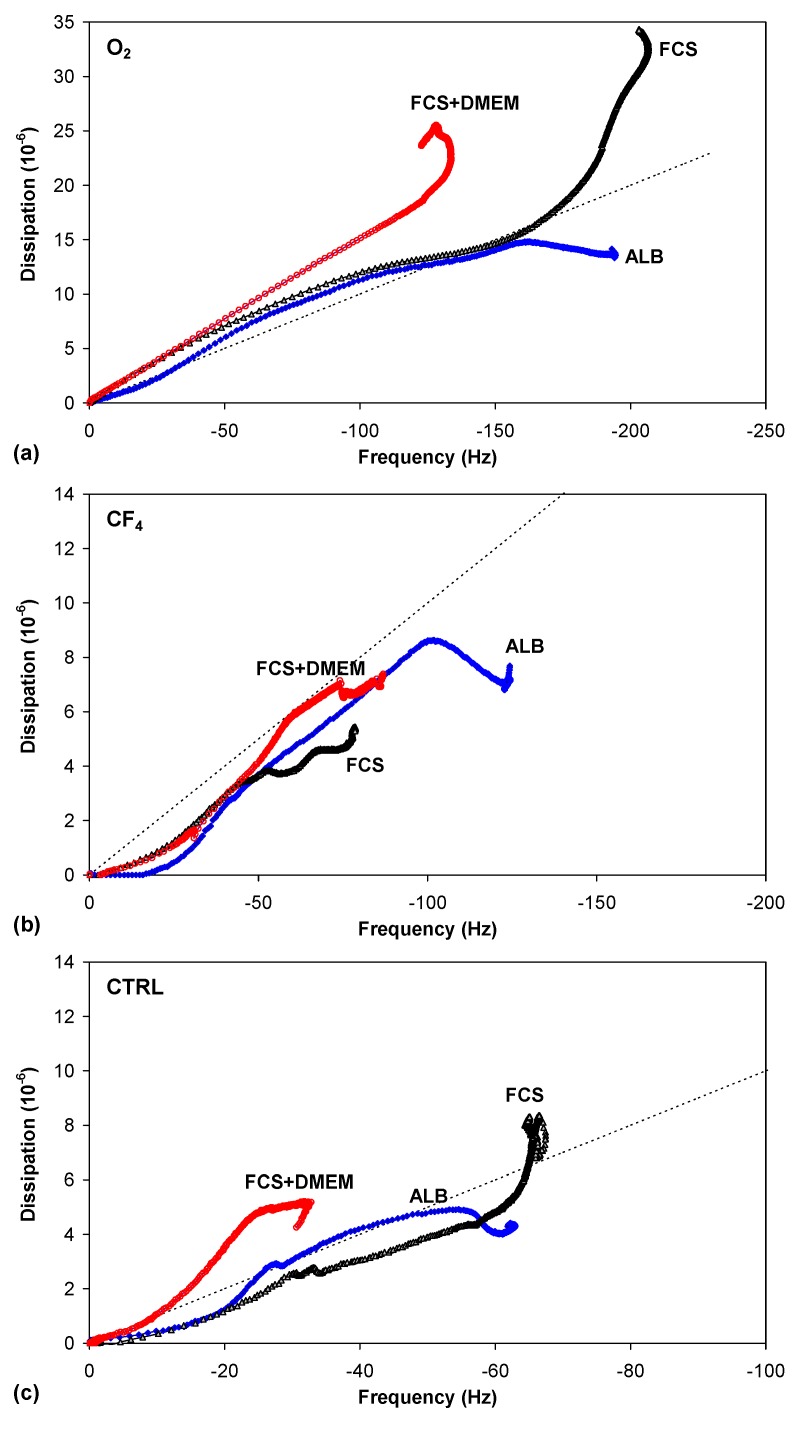
Δ*D* = f(Δ*f*) function for protein adsorption on plasma-treated PET substrates for albumin (**a**), FCS (**b**) and FCS with DMEM (**c**).

Figure 13 plots the dissipation change *versus* frequency change (Δ*D* = f(Δ*f*) plot), which can provide important information about the softness/rigidity of the protein layer formed during adsorption. A lower slope indicates a more dense and rigid layer, while a higher slope indicates the formation of a softer and more dissipating layer [26]. For the oxygen plasma, at the beginning, the spacing between the data points is greater, whereas the data points later become closertogether. This indicates faster adsorption kinetics at the beginning [25]. Additionally, the adsorption is uniform at the beginning, whereas the slope of the adsorption curve changes later (Figure 13a). For FCS and FCS/DMEM, the slope increased because of the faster deposition of mass, which might be caused by a change in conformation or even by multilayer adsorption. By contrast, for albumin, the slope decreased afterwards. Figure 13a also shows that all of the curves are positioned close together at the beginning. Shortly thereafter, the curve for FCS/DMEM turns away, while the curves for FCS and ALB remain very close for a longer time before finally turning away in opposite directions. 

The slope of the curve for the CF_4_ plasma treatment (Figure 13b) is much less steep, indicating a more densely adsorbed layer, in contrast to the oxygen plasma treatment, which has a very steep curve, indicating a more loosely bound layer. Thus, for the oxygen plasma treatment, the curve is always above the dotted line, showing the so-called “soft-rigid” boundary. By contrast, for the CF_4_ plasma treatment, the adsorption curve is always below the dotted line. For the control sample (Figure 13c), the slope is occasionally very close to the “soft-rigid” boundary, but only lies above this boundary for FCS/DMEM.

**Figure 14:**
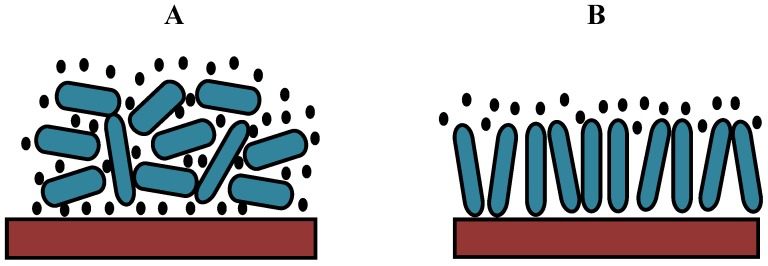
A hypothetical model for protein adsorption on an oxygen plasma-treated (hydrophilic) surface with trapped water molecules (**A**) and a CF_4_ plasma-treated (hydrophobic) surface (**B**).

On the basis of these results, we can propose a hypothetical model for protein adsorption on hydrophilic or hydrophobic surfaces (Figure 14). Albumin can be adsorbed in two different orientations: side-on or end-on [32,33]. Because the layer is more rigid for the CF_4_ plasma, the protein should be more closely packed, which can only be achieved in the end-on orientation. By contrast, the oxygen plasma results in a more loosely bound layer; thus, the protein is probably adsorbed in such a way that its configuration is similar to the side-on orientation (Figure 14). In this case, multilayer adsorption is likely to occur.

### 2.4. Cellular Response to Plasma-Treated Surfaces

**Figure 15:**
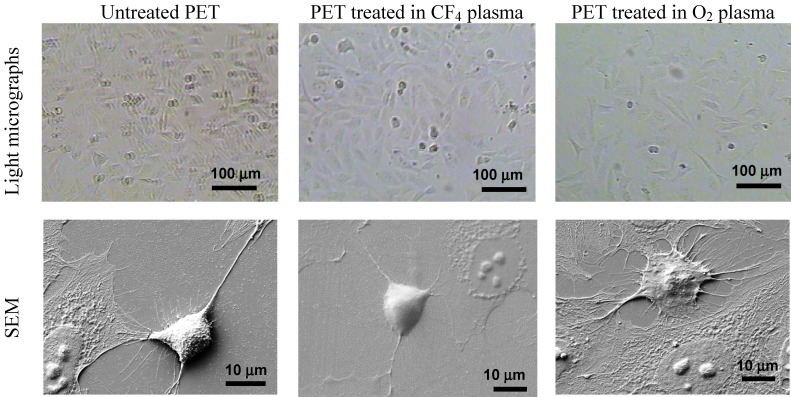
HOS cell adhesion and morphology on the PET polymer surface after plasma treatment, as measured using light microscopy and SEM analysis after 1 day of incubation.

Finally, to observe the influence of plasma treatment and protein adhesion on cell proliferation, we studied human osteosarcoma (HOS) cell adhesion. The characteristics of the cell adhesion and the morphology of HOS cells on different polymers were investigated with optical microscopy and SEM (Figure 15). The best adhesion of HOS cells was observed on the oxygen-plasma-treated samples, while there was no significant difference in cell adhesion between the control (untreated) and the fluorine-treated samples. Some differences between the samples can be observed in Figure 15.

For the control samples after 1 day of incubation, we observed spread cells that form some microvilli and active margins, allowing the cells to connect to each other. By contrast, on the oxygen plasma-treated polymers, we observed extensively spread cells. The majority of the cells pulled in their thinner margins, while others were already in mitosis. More microvilli and active margins, which connect between themselves and form an extracellular matrix for cells to adhere on the surface, were visible. Such cell morphology occurs in a suitable environment for cell growth, which we have provided with the oxygen-treated polymer surface. 

On the fluorine plasma-treated polymers after 1 day of incubation, the cells have a morphology similar to that of the control sample. Most cells are spread with a flattened nuclear region and are relatively free of microvilli, while some are similar to the spread cells observed in the control samples and are slightly rounded with raised nuclear regions.

**Figure 16:**
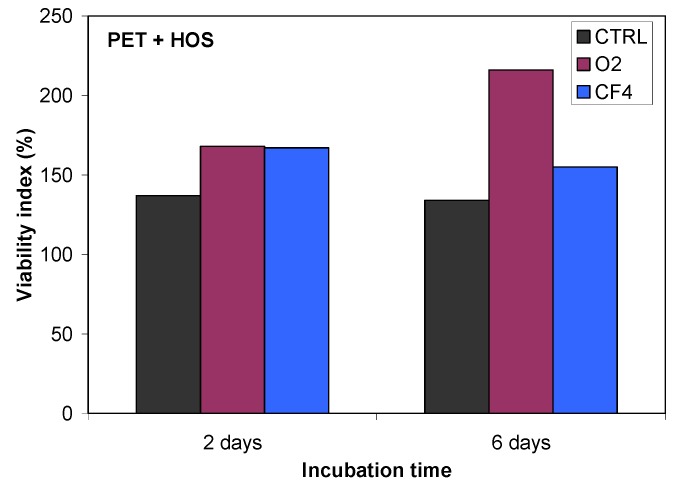
Proliferation of HOS cells on untreated and plasma-treated PET polymers, as measured using an MTT assay.

Plasma-treated samples were also tested for cell adhesion and proliferation (Figure 16), and a substantial difference in cell adhesion was found on the plasma-treated surfaces. The results of the MTT ((3-(4,5-dimethylthiazol-2-yl)-2,5-diphenyltetrazolium bromide) colorimetric assay for HOS cell incubation after 2 and 6 days are shown in Figure 16. This figure shows the much better cell proliferation for the O_2_ plasma-treated PET sample treated in compared with that for the CF_4_ plasma-treated sample. As demonstrated by QCM, this finding can be explained by the different protein conformation on the surface and the different mass of adsorbed proteins. 

Our QCM results are consistent with our study of cell proliferation, where we have observed improved cell proliferation and adhesion on the oxygen plasma-treated surface relative to that in the control sample or the CF_4_ plasma-treated sample. The improved cell proliferation occurs because the oxygen plasma-treated surface promotes protein adsorption. Furthermore, proteins are likely to maintain their active form on a hydrophilic surface; it was reported that on a hydrophilic surface, proteins prefer to maintain their conformations, whereas on a hydrophobic surface, proteins denature [34].

### 2.5. Discussion of Cell- and Protein-Material Interactions

A similar study was performed by Kurniawan *et al*. [35]. They used O_2_ and CF_4_ plasmas to modify the surface properties of bacterial cellulose (BC) fibres and study the adhesion of fibroblasts and Chinese hamster ovary cells. Interestingly, they found better cell adhesion on the CF_4_ plasma-treated surface than on the surface treated with O_2_ plasma. This was explained by the higher quantity of proteins adsorbed to the CF_4_ plasma-treated surface. Because those researchers used a different substrate material that has a fibrous structure (and not a foil) and different cells, it is difficult to correlate their work with ours. Furthermore, reports from some other authors support our finding that CF_4_ plasma is not optimal for cell adhesion [36,37]. In addition, Blackstone *et al*. found better cell adhesion of cancer cells on an air plasma-treated surface than on a CF_4_ plasma-treated surface [38]. 

Different authors may observe different results because of the complexity of plasma treatments. We have mentioned that plasma treatments can modify different surface properties of polymer materials, including the surface chemistry, polarity, roughness and hydrophilicity. It is difficult to perform a plasma treatment in a manner that changes only one surface property; therefore, it is not possible to determine explicitly which surface parameter is most important for cell and protein adhesion, e.g., the surface hydrophilicity can be varied using polar functional groups at the surface as well as by changing the surface roughness. Furthermore, surfaces with the same hydrophilicity may have different types of functional groups. For example, treating polymers with oxygen-containing plasma increases the surface hydrophilicity; however, osteoblast cells adhered to a pure oxygen plasma-treated surface, whereas there was less adhesion on the surface treated in sulphur dioxide plasma [39]. Jacobs *et al*. [39] and Griesser *et al*. [17] provide good reviews of plasma treatments and cell adhesion. Griesser reports that nitrogen-containing plasmas were used to improve cell adhesion because of the ability to induce amine groups on the surface [17,18]. Normally, the results showed an increase in cell adhesion with increasing nitrogen content, but some authors have also concluded that amine groups rather than the total nitrogen content is important. By contrast, other authors have found that amide groups appear to be the main surface chemical group that promotes cell adhesion [18]. The same problem arises with oxygen plasma, which also improves cell adhesion; however, it is also not clear which functional group promotes better adhesion of cells. Enhanced cell adhesion did not always correlate with the amount of oxygen at the surface. It appears that the surface chemistry must play an important role in surface interactions. Nevertheless, as shown in a review paper by Jacobs *et al*. [39], plasma treatments in O_2_, CO_2_, air and ammonia lead to improved cell elongation, attachment and/or proliferation; unfortunately, other plasmas, such as CF_4_, were not mentioned.

There are also interesting studies on the influence of surface hydrophilicity on cell adhesion. Lee *et al*. have performed a special corona treatment to make a wettability gradient along a polymer surface (from 97° to 48°) [40]. They found that moderately hydrophilic surfaces with a contact angle of 65° showed the best proliferation rate. By contrast, we can find reports of very good cell proliferation on very hydrophilic oxygen plasma-treated surfaces with a water contact angle below 10° [41]. Some authors, such as Dowling *et al*., have also studied the effect of surface wettability and roughness on cell proliferation [42]. Surfaces with a contact angle from 12° to 155° were obtained through a combination of modifying surface roughness (by grinding), deposition of siloxane coatings and surface fluorination. These researchers also reported optimum cell adhesion at 64°. The only problem is that they also changed two parameters: surface roughness and surface chemistry. When they changed only the surface roughness, cell adhesion increased with increasing surface roughness in the range of 19–2,365 nm. 

Surface roughness in combination with morphology can have an important influence on cell spreading. Therefore, there have been many previous attempts to study cell “contact-guidance” along specially patterned micro-structured surfaces. Good reviews of such studies have been published [43,44]. In recent years, nano-rough surfaces have been used to study cell adhesion. Besenbacher *et al*. provide a good review of the influence of nanoscale surface topography with surface features smaller than 100 nm [45]. From the review, it is obvious that nano-rough surfaces change protein conformations. Proteins with dimensions of the same order as the surface are not conformationally altered, while proteins with dimensions much smaller or larger than the surface roughness are conformationally altered upon adsorption. There appears to be no general trend in the amount of adsorbed proteins because different results were obtained. The same is true for cell proliferation. Some authors found enhanced cell proliferation and sometimes the cells were aligned along the nanogrooves, but some authors also found reduced cell adhesion. The type of cell appears to play an important role. Furthermore, the detailed shape of the nanofeatures may be important. Cells will normally align along the groves, but other topographical features, such as wells, pits or pillars, do not result in such a clear cell alignment [43]. We can only conclude that surface wettability, roughness and chemistry must be optimised for different polymer materials and different cell types.

## 3. Experimental

### 3.1. Plasma Treatment

Samples of poly(ethylene terephthalate) (PET) polymer (from Goodfellow Cambridge Ltd., Huntingdon, UK) in the form of thin disks with a thickness of 0.25 mm and diameter of 10 mm were exposed to oxygen (O_2_) or tetrafluoromethane (CF_4_) plasma. The plasma was created in a glass discharge tube with a diameter of 4 cm and length of approximately 60 cm. A rather uniform glow discharge was created within an RF coil, which was 15 cm long. The coil was connected to an RF generator operating at a frequency of 27.12 MHz with a nominal power of approximately 200 W. Commercially available oxygen or tetrafluoromethane was leaked into the glass tube on one side, while the other side was continuously pumped with a two-stage rotary pump. Continuous pumping allowed for rapid removal of any reaction products that might otherwise accumulate in the plasma reactor and distort the original gas composition. Samples of PET polymer were treated in plasma at a floating potential for 30 s. Samples were treated either in O_2_ plasma to make the surface hydrophilic or in CF_4_ plasma to make it hydrophobic.

### 3.2. Protein Adsorption

To study the kinetics of protein adsorption to plasma-treated surfaces, the samples were incubated in various protein solutions that are used as cell-culture medium. The following solutions were used: (i) 1% albumin (Sigma Aldrich Co., Taufkirchen, Germany) solution; (ii) 10% foetal calf serum (FCS) and (iii) DMEM supplemented with 10% FCS serum. DMEM (Dulbecco’s Modified Eagle’s Medium) contains amino acids, salts (calcium chloride, potassium chloride, magnesium sulphate, sodium chloride and monosodium phosphate), glucose and vitamins (folic acid, nicotinamide, riboflavin and B12). By contrast, FCS contains a rich variety of proteins, and a major component is albumin.

### 3.3. Quartz Crystal Microbalance (QCM)

The mass of the proteins adsorbed to the untreated polymer or the plasma-treated PET polymer was studied for various incubation times. The adsorption kinetics of the proteins was determined using a quartz crystal microbalance with a dissipation unit, QCM-D (Model E4, QSense AB, Göteborg, Sweden). The QCM measured the mass of the protein adsorbed on a PET film that was spin-coated onto a quartz crystal sandwiched between two electrodes. The electrodes were connected to a power supply that caused the crystal to oscillate at its fundamental resonance frequency and several overtones. The resonance frequency was disturbed by the addition or removal of mass. The frequency shift induced by a mass change was calculated using the Sauerbrey model [16,18]. Thus, the adsorption rate of proteins *versus* incubation time was measured. In addition to measuring the frequency, the dissipation was measured. The dissipation is a parameter quantifying the damping in the system, and it is related to the sample’s viscoelastic properties.

### 3.4. X-Ray Photoelectron Spectroscopy (XPS)

The surface functionalisation of the polymer samples after plasma treatment was studied by high-resolution X-ray photoelectron spectroscopy. The samples were exposed to air for a few minutes after the plasma treatment and then mounted in a TFA XPS instrument (Physical Electronics Inc., Chanhassen, MN, US). The base pressure in the XPS analysis chamber was approximately 6 × 10^−8^ Pa. The samples were excited with X-rays over a 400-µm spot area with monochromatic Al K_α__1,2_ radiation at 1486.6 eV. The photoelectrons were detected with a hemispherical analyser positioned at an angle of 45° with respect to the normal sample surface. The energy resolution was approximately 0.5 eV. Survey-scan spectra were acquired at a pass energy of 187.85 eV, while for C1s, individual high-resolution spectra were taken at a pass energy of 23.5 eV with a 0.1 eV energy step. Because the samples were insulators, an additional electron gun was used to allow for surface neutralisation during the measurements. All spectra were referenced to the main C1s peak of the carbon atoms, which was assigned a value of 284.8 eV. The concentration of the different chemical states of carbon in the C1s peak was determined by fitting the curves with symmetrical Gauss-Lorentz functions. The spectra were fitted using MultiPak v8.1c software (Ulvac-Phi Inc., Kanagawa, Japan, 2006) from Physical Electronics, which was supplied with the spectrometer. 

### 3.5. Atomic Force Microscopy (AFM)

An AFM (Solver PRO, NT-MDT, Moscow, Russia) was used to characterise the surface topography of the samples. All measurements were performed in semi-contact mode using golden silicon probes NSG10 tips (NT-MDT, Limerick, Ireland) with a resonance frequency of 140–390 kHz and force constant of 3.1–37.6 N/m. The images were 2 × 2 µm^2^. 

### 3.6. Cell Adhesion, Growth and Viability (MTT Assay)

Human osteosarcoma cells (HOS) were seeded at 2 × 10^4^ cells in 100 μL of medium on the upper side of the polymers (concentration: 2.55 × 10^4^ cells/cm^2^) and left for 3 h to attach before the addition of medium to cover the polymer discs [19,20]. Cells were plated in DMEM supplemented with 10% FCS (foetal calf serum) and left to grow on polymer discs at 37 °C in a humidified atmosphere of 5% CO_2_. Triplicates of the cultures for each time and treatment were prepared for adhesion and cell viability assays. 

Cell adhesion was monitored daily, and micrographs of samples on the different polymer surfaces were taken after the 1st and 6th days of culturing. The MTT-related colorimetric assay (EZ4U; Biomedica GmbH, Wien, Austria) was used to determine cell growth and viability, according to the manufacturer's instructions and Jaganjac *et al*. [20]. This method is based on the fact that living cells are capable of reducing the less-coloured tetrazolium salts into intensely coloured formazan derivatives. This reduction process requires functional mitochondria, which are inactivated within a few minutes after cell death.

Briefly, after the 1st and 6th days of HOS cell culture on the different polymer surfaces, the medium was removed and 1 mL of fresh Hanks’ Balanced Salt Solution (HBSS) and 100 μL of the tetrazolium agent were added to each culture. After 2 h of incubation, the supernatants were transferred into 96-well plates and measured in a microplate reader (Easy-Reader 400 FW, SLT Lab Instruments GmbH, Salzburg, Austria) at 450/620 nm.

### 3.7. Scanning Electron Microscopy (SEM)

Cell adhesion was also monitored with a scanning electron microscope. In this case, a rather short cell incubation time of 4 h was chosen because we wanted to capture the initial moments of the cell adhesion. Prior to SEM measurements, the samples were dehydrated in a sequence of alcohols and then vacuum dried and finally covered with a thin layer of gold. For gold evaporation PECS instrument (Model 682) from Gatan GmbH (München, Germany) was used. SEM analyses were performed at two magnifications, 250× (not shown) and 1,500× using a JEOL JSM-840 Scanning Electron Microscope (JEOL, Tokyo, Japan). 

## 4. Conclusions

The adsorption rate of proteins on the plasma-treated PET substrates was studied using the QCM-D technique. The source of proteins was the cell-culture medium, which is used to support the growth of cells. PET was treated either in oxygen plasma to make the surface hydrophilic or in CF_4_ plasma to make the surface hydrophobic. At low protein incubation time, XPS results indicated a similar concentration of proteins on both plasma-treated surfaces in comparison to the pristine untreated sample. By contrast, for longer incubation times, the differences between the plasma-treated samples and the untreated sample diminished, as detected by XPS. Compared to XPS, QCM can provide much better information regarding the adsorption kinetics of proteins. The adsorption rate was fastest for the oxygen plasma-treated sample and slowest for the control sample. The protein adsorption on the oxygen plasma-treated surface caused the maximum change in frequency as well as in dissipation, indicating maximum adsorption. By contrast, the control sample caused the minimum protein adsorption. Thus, the mass of the adsorbed proteins was highest for the oxygen plasma-treated sample, lowest for the control sample, and somewhere in between for the fluorine plasma-treated sample. The results clearly demonstrate that hydrophilic surfaces can also adsorb a large amount of proteins, in our case twice as much as hydrophobic surfaces. Therefore, such hydrophilic surfaces caused better cell proliferation and adhesion compared with the more hydrophobic surfaces. 
